# Arming oncolytic M1 virus with gasdermin E enhances antitumor efficacy in breast cancer

**DOI:** 10.1016/j.isci.2024.111148

**Published:** 2024-10-16

**Authors:** Xiao-yu Chen, Ying Liu, Wen-bo Zhu, Shu-hao Li, Song Wei, Jing Cai, Yuan Lin, Jian-kai Liang, Guang-mei Yan, Li Guo, Cheng Hu

**Affiliations:** 1 Department of Urology, The Third Affiliated Hospital of Sun Yat-sen University, 600# Tianhe Road, Guangzhou, Guangdong 510630, China; 2 Department of Infectious Diseases, The Third Affiliated Hospital of Sun Yat-sen University, 600# Tianhe Road, Guangzhou, Guangdong 510630, China; 3 Departments of Pharmacology, Sun Yat-sen University, No. 074, Zhongshan Second Road, Guangzhou 510080, China; 4 Advanced Medical Technology Center, The First Affiliated Hospital-Zhongshan School of Medicine, Sun Yat-sen University, Guangzhou, China; 5 Key Laboratory of Human Microbiome and Elderly Chronic Diseases, Ministry of Education, Guangzhou, China; 6 Collaborative Innovation Center for Cancer Medicine, Guangzhou, China

**Keywords:** Oncology, Therapy, Immunology

## Abstract

Pyroptosis, driven by the N-terminal domain of gasdermin proteins (GSDM), promotes antitumor immunity by attracting lymphocytes to the tumor microenvironment (TME). However, current pyroptosis-inducing therapies like drug injections and phototherapy are limited to localized treatments, making them unsuitable for widespread or microscopic metastatic lesions. This study engineered oncolytic M1 viruses (rM1-mGSDME_FL and rM1-mGSDME_NT) to selectively deliver GSDME to tumor cells. These modified viruses enhanced tumor cell death in breast cancer models, suppressed tumor growth, extended survival in mice, and boosted immune cell infiltration, demonstrating significant anticancer potential through pyroptosis induction.

## Highlights

• Arming M1 virus with GSDME enhances pyroptosis in breast cancer cells
• rM1-mGSDME_FL and rM1-mGSDME_NT suppress tumor growth and prolong survival in mice
• Enhanced CD8^+^ T cells and NK cells infiltration in the tumor microenvironment
• Engineered M1 viruses induce significant immune responses through pyroptosis

## Graphical abstract

**Figure:**
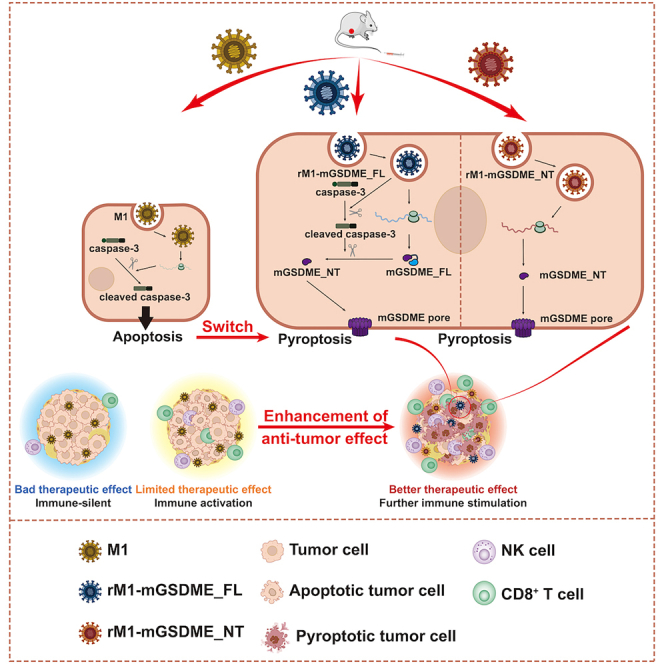

## Introduction

Oncolytic viruses (OVs) represent a promising class of cancer therapeutics that selectively replicate in and induce apoptosis of cancer cells while sparing normal tissues.^1^ Most OVs are genetically engineered to enhance their tumor-targeting specificity, selective replication, lytic potential, and ability to stimulate the host’s antitumor immune response.^2^ The M1 virus, isolated from Culex in Hainan Island, China, is a Getah-like virus with a single-stranded positive-sense RNA genome.^3^ Our previous studies identified the M1 virus act as a novel anticancer agent. We first discovered that the oncolytic M1 virus could induce apoptosis in rat malignant glioma cell lines (C6).^4^ M1 virus exhibited robust oncolytic effects in several human cancer cell lines. Notably, M1 virus generated a significant oncolytic effect in tumor cells lacking zinc-finger antiviral protein (ZAP), inducing endoplasmic reticulum stress (ER-stress) and leading to enhanced oncolysis.^5^

After assessing the safety of the M1 virus in normal cell lines, mice, and non-human primates, our focus shifted toward maximizing the oncolytic potential of the M1 virus. Several Key molecular pathways involved in M1 replication and oncolysis were identified, including inhibition of the mevalonate pathway, loss of the IRE1α-autophagy pathway, activation of the cAMP pathway, and inhibition of valosin-containing protein (VCP).^6^^,^^7^^,^^8^^,^^9^^,^^10^These findings highlighted the significant enhancement of the M1 virus’s antitumor effects through the use of various small molecule compounds, providing reliable adjuvants and molecular markers for subsequent clinical studies. However, we have observed low sensitivity to the M1 virus in some tumor cell lines and clinical tumor biopsies, where small molecule compounds fail to sensitize these cells. Thus, enhancing the oncolytic efficacy of the M1 virus while maintaining its high tumor specificity and broadening its therapeutic scope remains a critical scientific challenge for future research.

Apoptosis-related caspases have long been recognized as critical regulators of programmed cell death. Traditionally, these caspases were associated with the induction of apoptosis, a process essential for maintaining cellular homeostasis and eliminating damaged or diseased cells.^11^ However, emerging research has revealed a novel role for these caspases in triggering a different form of cell death known as pyroptosis.^12^ Pyroptosis is a newly discovered and validated form of programmed cell death. Research has shown that gasdermin (GSDM) family proteins act as executioners in pyroptosis.^13^^,^^14^ The GSDM family consists of several members, with GSDMD and GSDME being the most extensively studied pyroptotic effectors. GSDMD is activated through cleavage by inflammatory caspases, such as caspase-1, 4, 5, and 11, while GSDME can be cleaved by caspase-3, releasing the N-terminal domain to punch holes in the cell membrane, causing cellular lysis and the subsequent release of inflammatory mediators.^15^^,^^16^ Pyroptosis is characterized by continuous cell swelling until the cell membrane ruptures, typically accompanied by the release of pro-inflammatory cytokines (such as IL-1β and IL-18) and cellular contents (such as HMGB1 and LDH), triggering a robust inflammatory response.^12^^,^^17^ Tumor pyroptosis can be triggered through multiple methods, including intratumoral injection, phototherapy, ultrasound therapy, and intravenous administration of drugs or nanomedicines,^18^^,^^19^^,^^20^^,^^21^ all of which are local therapies and are not suitable for multiple metastatic lesions or microscopic lesions.

Breast cancer is one of the most prevalent and lethal cancers among women worldwide. Despite advancements in detection and treatment, breast cancer continues to pose significant health challenges due to its heterogeneity and potential for metastasis.^22^^,^^23^ Triple-negative breast cancer (TNBC), in particular, is unresponsive to hormonal and HER2-targeted therapies, resulting in limiting treatment options and leading to poorer prognosis.^24^^,^^25^

In this study, we aimed to develop an efficient system to induce pyroptosis in breast cancer cells by using the oncolytic M1 virus as a delivery vehicle for GSDME. The activation of GSDME relies on cleavage by caspase-3, and M1 infection induces cleaved caspase-3 in tumor cells.^5^ However, there is uncertainty regarding whether M1 replication generates sufficient cleaved caspase-3 to fully activate GSDME_FL. To address this, we constructed two armed viruses: rM1-mGSDME_FL, which requires caspase-3 activity for GSDME activation, and rM1-mGSDME_NT, which directly expresses the active NT fragment, bypassing the need for caspase-3 cleavage. Both rM1-mGSDME_FL and rM1-mGSDME_NT were designed to enhance M1’s antitumor efficacy *in vitro* and *in vivo* by inducing pyroptosis. The comparison between rM1-mGSDME_FL and rM1-mGSDME _NT will provide insight into which construct more effectively induces pyroptosis and contributes to antitumor activity.

## Results

### Replication kinetics and cytopathic effects of armed M1 viruses

**Figure 1:**
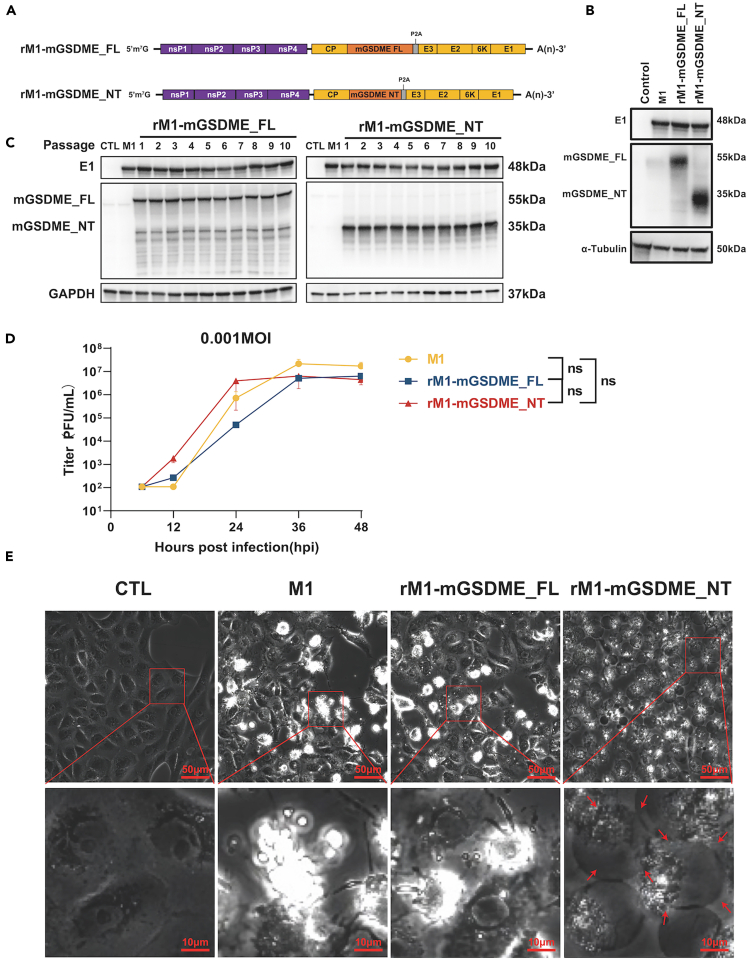
Characterization of rM1-mGSDME constructs and their expression (A) Schematic representation of the two rM1-mGSDME constructs. rM1-mGSDME_FL (Full-Length) includes the complete mGSDME sequence. rM1-mGSDME_NT (N-terminal) includes only the N-terminal domain of mGSDME. (B) Western blot analysis of E1, mGSDME_FL, mGSDME_NT, and α-tubulin (loading control) in control, M1, rM1-mGSDME_FL, and rM1-mGSDME_NT samples. (C) Western blot analysis of E1, mGSDME_FL, mGSDME_NT, and GAPDH (loading control) in various passages (1–10) of cells transfected with rM1-mGSDME_FL and rM1-mGSDME_NT constructs. (D) The cells were infected with each virus at an MOI of 0.001, and viral titers were measured at 0, 12, 24, 36, and 48 h post-infection (hpi) (Data are represented as mean ± SEM). (E) Morphology of Vero cells 24 h post-infection with M1, rM1-mGSDME_FL, and rM1-mGSDME_NT compared to the control group, Scale bar: 50 μm for the upper panel, 10 μm for the lower panel.

The armed OVs rM1-mGSDME_FL and rM1-mGSDME_NT, incorporating full-length and N-terminal fragments of mGSDME into the M1 viral genome, were constructed through genetic engineering techniques (Figure 1A). We obtained infectious primary armed M1 viruses (rM1-mGSDME_FL and rM1-mGSDME_NT) in Vero cells using *in vitro* transcription and transfection. Western blot analysis showed successful expression of the viral structural protein E1 and the mGSDME_FL or mGSDME_NT proteins in Vero cells infected with the respective viruses, demonstrating successful incorporation and expression of the exogenous mGSDME genes (Figure 1B). Additionally, continuous passage of these viruses in Vero cells demonstrated stable expression of both the E1 protein and the respective mGSDME proteins across 10 passages, confirming consistent gene expression during viral replication (Figure 1C). These results validate the successful construction and stable expression of the exogenous mGSDME genes in the armed OVs.

To assess the impact of genetic engineering on viral replication, we analyzed the replication kinetics and cell morphology of Vero cells infected with M1, rM1-mGSDME_FL, and rM1-mGSDME_NT at an MOI of 0.001. The replication curves indicated that rM1-mGSDME_FL replicated slightly slower than the parental M1 virus, while rM1-mGSDME_NT replicated slightly faster (Figure 1D). Both armed viruses reached similar plateau titers to the parental M1 virus by 48 h post-infection. Examination of cytopathic effects (CPE) at 24 h post-infection revealed that cells infected with M1 and rM1-mGSDME_FL exhibited similar morphological changes, characterized by cell shrinkage. In contrast, cells infected with rM1-mGSDME_NT showed distinct ballooning and bubbling, hallmark signs of pyroptosis (Figure 1E). These results demonstrate the successful construction and rescue of the armed viruses rM1-mGSDME_FL and rM1-mGSDME_NT, with differential impacts on viral replication and CPE in Vero cells.

### Pyroptosis induction and cytotoxic effects by armed M1 viruses in breast cancer cells

**Figure 2:**
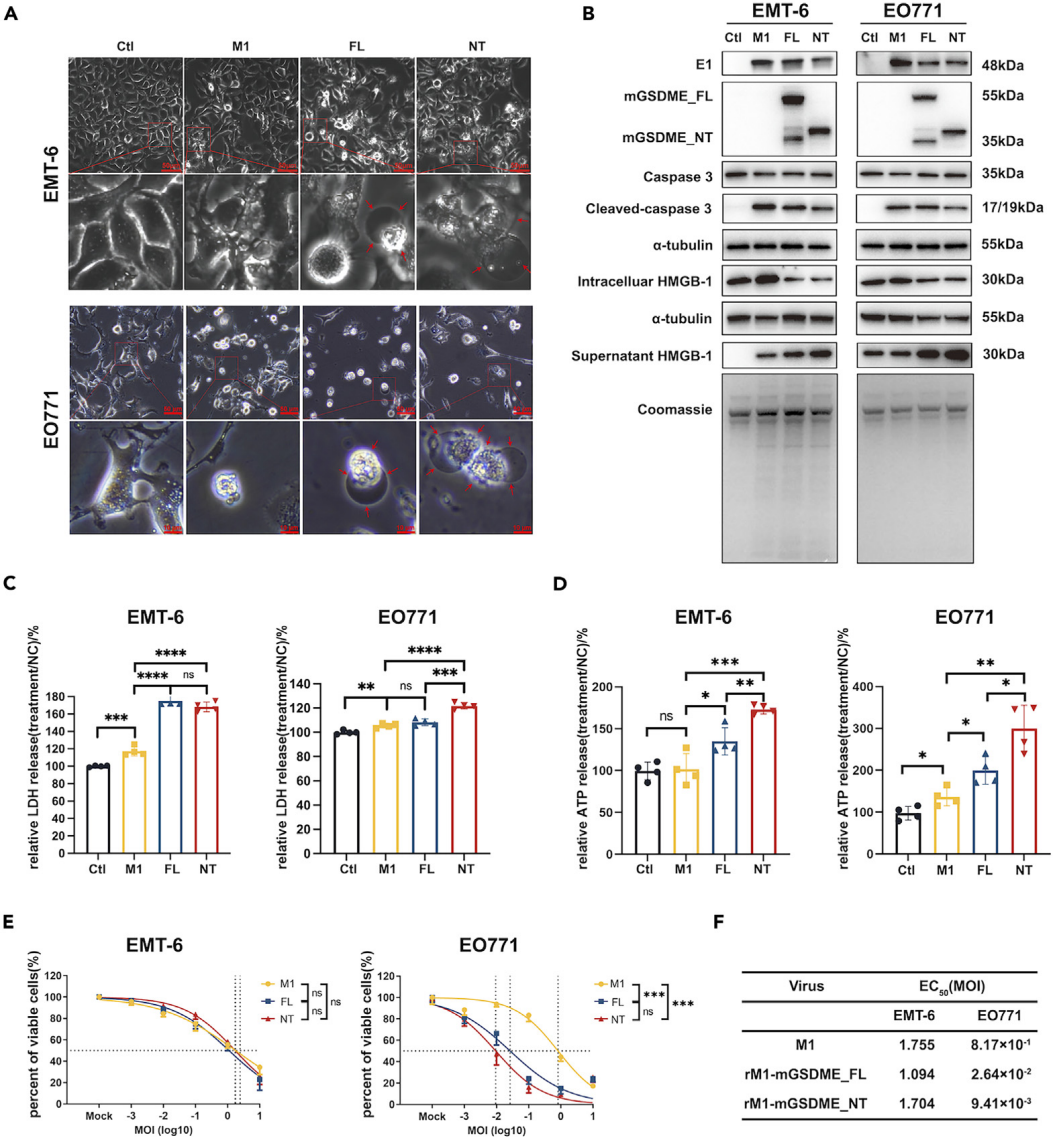
Induction of pyroptosis by armed oncolytic M1 viruses in breast cancer cell lines (A) Morphological changes were observed in EMT-6 cells (24 h post-infection) and EO771 cells (48 h post-infection) following treatment with control (Ctl), M1, rM1-mGSDME_FL (FL), and rM1-mGSDME_NT (NT). The images at 50 μm(upper panel) and 10 μm(lower panel) magnifications show significant pyroptotic features such as membrane swelling and bubbling (indicated by red arrows) in cells infected with FL and NT. (B) Western blot analysis was performed to detect the expression of viral protein E1, mGSDME_FL, mGSDME_NT, caspase 3, cleaved-caspase 3, intracellular and supernatant HMGB1, and α-tubulin in EMT-6 and EO771 cells infected with Ctl, M1, FL, and NT. Protein samples were collected at 24 h post-infection for EMT-6 cells and 48 h for EO771 cells. (C and D) Relative LDH and ATP release were measured in EMT-6 and EO771 cells 24 h post-infection following treatment with Ctl, M1, FL, and NT at an MOI of 1.0 (log10) (Data are represented as mean ± SEM). (E) Dose-response curves showing the percentage of viable cells infected with M1, FL, and NT at various MOIs (log10) (Data are represented as mean ± SEM). (F) EC50 values (MOI) for M1, FL, and NT in EMT-6 and EO771 cell line.

The antitumor activity of armed oncolytic M1 viruses was validated *in vitro* by infecting EMT-6 and EO771 cell lines. Morphological analysis of EMT-6 and EO771 cells (Figure 2A) revealed that both rM1-mGSDME_FL and rM1-mGSDME_NT induced significant pyroptosis, characterized by extensive membrane blebbing, followed by ballooning and eventual loss of membrane integrity. In contrast, the control (Ctl) group exhibited well-defined cell edges with no signs of swelling or membrane rupture and the M1 group showed features of apoptosis, including cell fragmentation and the formation of classic apoptotic bodies. Western blot analysis showed that infection with rM1-mGSDME_FL and rM1-mGSDME_NT led to the expression of viral protein E1 and the corresponding mGSDME FL/NT fragments, accompanied by increased levels of cleaved caspase-3, all indicating the successful replication of virus vectors (Figure 2B). Additionally, more pyroptosis-related markers were assessed in EMT-6 and EO771 cells following treatment with M1 and rM1 viruses. Western blot analysis indicated that in both the FL and NT groups, supernatant HMGB1 levels increased, while intracellular HMGB1 levels decreased (Figure 2B). LDH and ATP release assay results showed significant increases in LDH and ATP expression in the FL and NT groups compared to the M1 group, with statistically significant differences (Figures 2C and 2D). The cytotoxic effects of rM1 on EMT-6 and EO771 cells were subsequently investigated *in vitro*. MTT assay results indicated that in EMT-6 cells, cell viability in both the FL and NT groups was comparable to that of the M1 group, with overlapping cytotoxicity curves and no statistically significant differences in EC50. However, in EO771 cells, the FL and NT groups exhibited stronger cytotoxic effects than the M1 group at lower viral titers (MOI <0.1), as evidenced by a leftward shift in the cytotoxicity curves and a statistically significant reduction in EC50 (Figures 2E and 2F). These results suggest that the successful expression of mGSDME_FL and mGSDME_NT induces pyroptosis in both EMT-6 and EO771 cells.

### Enhanced antitumor efficacy of armed oncolytic M1 viruses in EMT-6 tumor model

**Figure 3:**
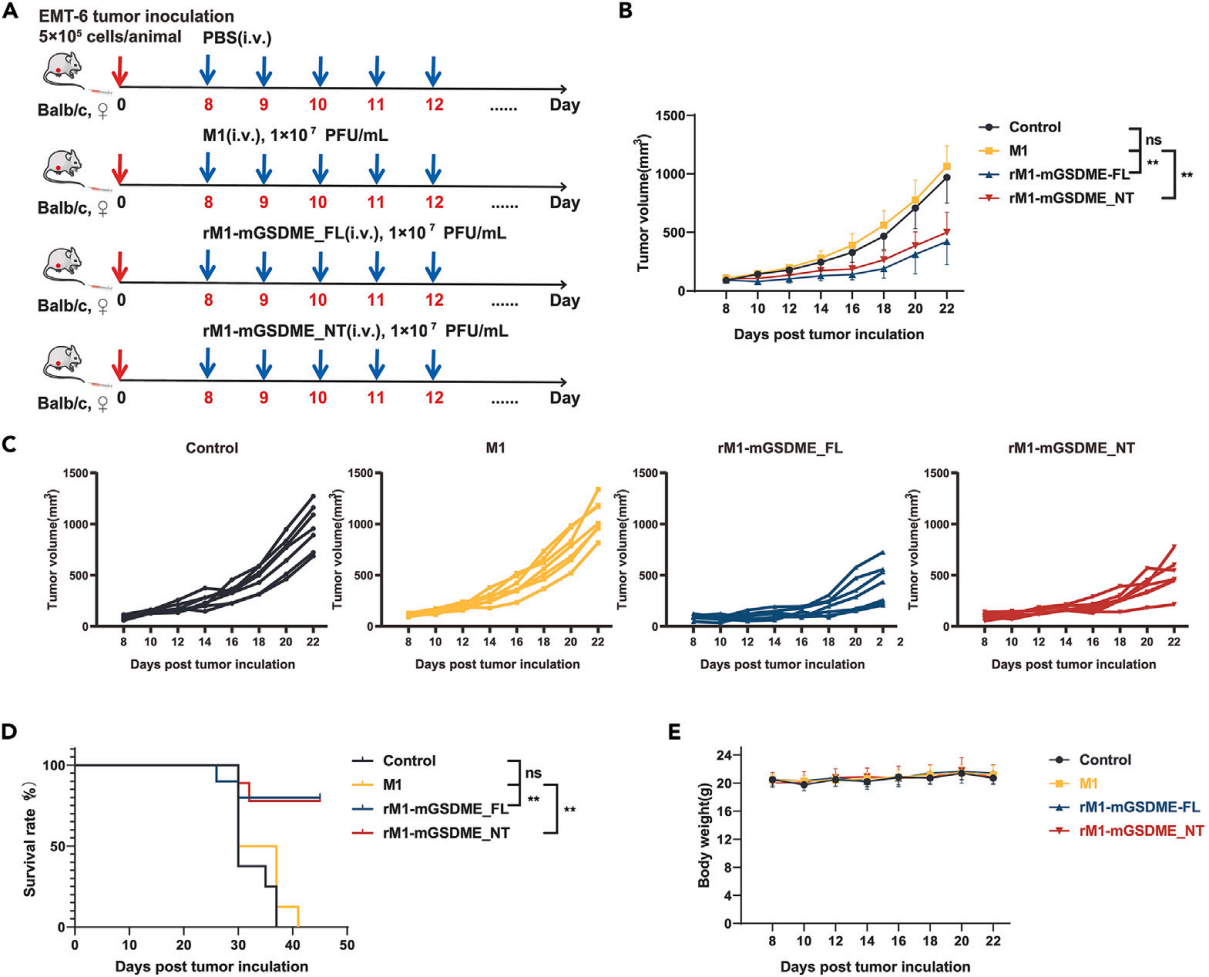
Antitumor effects of armed oncolytic M1 viruses in EMT-6 tumor-bearing mice (A) Schematic representation of the experimental setup. EMT-6 tumor cells were injected subcutaneously into the left flank of Balb/c mice. Seven days post-tumor implantation, mice received daily tail vein injections of PBS (control), M1, rM1-mGSDME_FL, or rM1-mGSDME_NT for five consecutive days. (B) Tumor growth curves showing the mean tumor volume (mm³) over time in mice treated with PBS, M1, rM1-mGSDME_FL, or rM1-mGSDME_NT. ∗∗*p* < 0.01, indicating significant differences between rM1-mGSDME_FL/rM1-mGSDME_NT and the M1 group (Data are represented as mean ± SEM). (C) Individual tumor growth curves for each treatment group, highlighting the variability in tumor volume among mice. (D) Kaplan-Meier survival curves for EMT-6 tumor-bearing mice treated with PBS, M1, rM1-mGSDME_FL, or rM1-mGSDME_NT. ∗∗*p* < 0.01, indicating significantly extended survival in mice treated with rM1-mGSDME_FL or rM1-mGSDME_NT compared to the M1 group. (E) Body weight measurements of EMT-6 tumor-bearing mice over the course of the study, showing no significant changes in body weight among the different treatment groups (Data are represented as mean ± SEM).

To evaluate the oncolytic effects of rM1-mGSDME_FL and rM1-mGSDME_NT *in vivo*, we established a mouse subcutaneous model by injecting EMT-6 tumor cells into the left flank of Balb/c mice. Seven days post-implantation, the mice received daily tail vein injections of PBS, M1, rM1-mGSDME_FL, or rM1-mGSDME_NT for five consecutive days (Figure 3A). Tumor volume measurements revealed that rM1-mGSDME_FL and rM1-mGSDME_NT significantly inhibited tumor growth compared to the M1 and control groups (Figure 3B). Individual tumor growth curves further confirmed the consistent antitumor activity of the armed viruses (Figure 3C). Kaplan-Meier survival analysis showed that mice treated with rM1-mGSDME_FL and rM1-mGSDME_NT had significantly extended survival compared to those treated with M1 or PBS (Figure 3D). Throughout the observation period, there were no significant changes in body weight among the treatment groups (Figure 3E), indicating no major adverse effects from the treatments. These results demonstrate the enhanced antitumor efficacy of rM1-mGSDME_FL and rM1-mGSDME_NT in the EMT-6 tumor model compared to the parental M1 virus.

### Immune cell infiltration in the tumor microenvironment enhanced by armed M1 viruses

**Figure 4:**
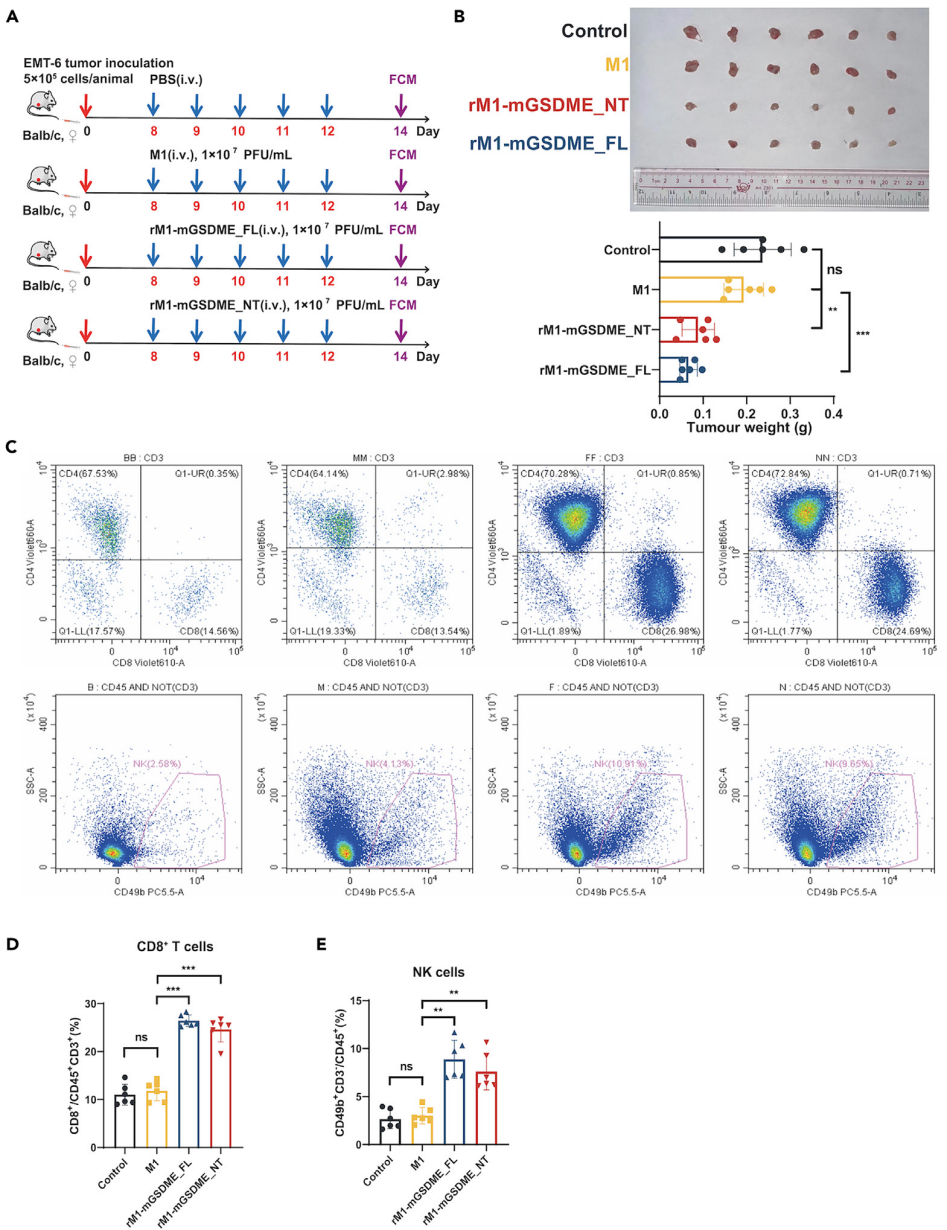
Antitumor and immune response effects of armed oncolytic M1 viruses in EMT-6 tumor-bearing mice (A) Schematic representation of the experimental setup. EMT-6 tumor cells were injected subcutaneously into the left flank of Balb/c mice. Seven days post-tumor implantation, mice received daily tail vein injections of PBS (control), M1, rM1-mGSDME_FL, or rM1-mGSDME_NT for five consecutive days. Tumors and immune cells were analyzed on day 14 post-tumor implantation by flow cytometry (FCM). (B and C) Flow cytometry analysis of tumor-infiltrating lymphocytes showing representative dot plots for CD8^+^ T cells (CD8^+^CD3^+^CD45^+^) and NK cells (CD3^−^CD49b^+^CD45^+^) in the tumors (Data are represented as mean ± SEM). (D and E) Quantification of CD8^+^ T cells (D) and NK cells (E) in the tumor microenvironment. Both rM1-mGSDME_FL and rM1-mGSDME_NT groups showed a significant increase in T cells and NK cell infiltration compared to the control and M1 groups (Data are represented as mean ± SEM).

Given the critical role of immune cell infiltration in the TME for antitumor immunity and the ability of pyroptosis to recruit substantial lymphocytes to the TME, we investigated the changes in immune cells within the TME following treatment with armed OVs. In the TNBC EMT-6 model, tumors from mice treated with M1, rM1-mGSDME_FL, or rM1-mGSDME_NT were subjected to immune flow cytometry analysis (Figure 4A). Consistent with the results from previous experiments, rM1-mGSDME_FL and rM1-mGSDME_NT treatments significantly reduced EMT-6 tumor weight (Figure 4B). Immunoflow cytometry analysis of the TME (Figure 4C) revealed a significant increase in the infiltration of CD8^+^ T cells (Figure 4D) and NK cells (Figure 4E) in tumors treated with rM1-mGSDME_FL and rM1-mGSDME_NT compared to control and M1 groups. These results indicate that treatment with rM1-mGSDME_FL and rM1-mGSDME_NT enhances lymphocyte infiltration, contributing to their superior antitumor efficacy in the EMT-6 model.

## Discussion

Cancer is one of the major global health issues, causing nearly 10 million deaths in 2020.^26^ While chemotherapy drugs exhibit strong cytotoxicity toward cancer cells, their limitations include drug resistance and off-target effects, which sometimes fail to provide sufficient antitumor protection and prevent tumor recurrence.^27^ Furthermore, many cancer cells have developed various methods to evade immune surveillance.^28^ In response, novel strategies like oncolytic virus immunotherapy have emerged as promising alternatives.^29^ These therapies not only induce apoptosis and release more OVs to kill surrounding tumor cells but also expose the pathogen-associated molecular patterns (PAMPs) and damage-associated molecular patterns (DAMPs) of tumor cells. This process enhances and induces antitumor immunity by releasing new antigens from damaged cancer cells, helping the immune system recognize tumors, promote inflammatory responses, and trigger immunogenic cell death (ICD). Consequently, this rapidly attracts the host defense mechanisms’ attention, leading to subsequent antitumor immune responses.^30^^,^^31^^,^^32^ Additionally, the OVs themselves act as antigens, activating immune cells, and stimulating the production of inflammatory factors that help eliminate newly formed tumor tissue.^30^ Thus, the unique targeting capability, high-efficiency replicability, and safety profile of oncolytic therapy make it a promising new antitumor treatment. Despite these advantages and ongoing clinical trials, some tumor cell lines remain resistant to the oncolytic virus M1,^33^ indicating that the oncolytic virus M1 still has significant potential for enhancement.

Pyroptosis is a form of programmed cell death characterized by inflammation, mediated by the Gasdermin protein family. Specifically, the N-terminal domain of GSDME forms pores in the cell membrane, leading to cell lysis and the release of inflammatory cytokines.^13^^,^^14^ GSDME expression was frequently epigenetic silenced by methylation in several cancers, including breast cancers.^34^ This methylation-induced silencing of GSDME has been linked to poor prognosis and lower survival rates in breast cancer patients.^35^ In pharmacokinetic studies, the oncolytic virus M1 demonstrated stable pharmacodynamic properties when administered via tail vein injection,^36^ with good safety and tumor cumulative efficiency profiles.^37^ It selectively targets tumor cells that have low or absent expression of the intracellular factor ZAP and those expressing the cell membrane receptor MXRA8.^38^ This selective cytotoxicity makes the M1 virus an ideal vector for delivering the pyroptosis-inducing molecule GSDME. Therefore, we armed the oncolytic virus M1 with mGSDME and explored the potential of armed OVs rM1-mGSDME_FL and rM1-mGSDME_NT as promising therapeutic agents for the treatment of breast cancer.

In EMT-6 and EO771 breast cancer cells, both rM1-mGSDME_FL and rM1-mGSDME_NT induced significant pyroptosis, morphologically accompanied by balloon-like swelling and changes in cell membrane permeability. Western blot analysis further validated these findings by showing elevated levels of cleaved-caspase 3 in EMT-6 and EO771 cells infected with rM1-mGSDME_FL. The cleavage of caspase 3 indicates activation of the apoptotic pathway, which subsequently led to the cleavage of mGSDME_FL into its active N-terminal fragment.^39^ This cleavage event is crucial for executing pyroptosis, resulting the release of inflammatory substances, with a decrease in intracellular HMGB1 levels and an increase in extracellular LDH and ATP expression, providing clear evidence that the infection with rM1-mGSDME_FL effectively induced pyroptosis in the breast cancer cells.

Using the mouse subcutaneous EMT-6 tumor model, we observed that the armed OVs rM1-mGSDME_FL and rM1-mGSDME_NT exhibit superior antitumor efficacy compared to the parental M1 virus and control groups. Both engineered viruses significantly inhibited tumor growth and extended the survival of the treated mice, indicative of their potential as effective therapeutic agents against TNBC. The enhanced antitumor effects observed with rM1-mGSDME_FL and rM1-mGSDME_NT treatments were accompanied by a marked increase in the infiltration of CD8^+^ T cells and NK cells into the TME. This increased immune cell infiltration is indicative of a robust immune response, likely contributing to the superior antitumor efficacy of the armed viruses. CD8^+^ T cells and NK cells are critical components of the immune system’s ability to target and destroy tumor cells,^40^ and their presence in greater numbers within the TME suggests that the armed viruses are not only directly lysing tumor cells but also enhancing the body’s natural immune defenses against the tumor. The ability of the armed viruses to induce pyroptosis, a form of programmed cell death characterized by inflammation and immune activation, may explain the increased immune cell infiltration. Pyroptosis can release DAMPs, which act as signals to recruit and activate immune cells.^12^ This process likely creates a more immunogenic TME, facilitating the infiltration and activation of CD8^+^ T cells and NK cells, and thereby amplifying the antitumor immune response.

Immune checkpoint blockade therapies, such as anti-PD1 and anti-PD-L1, have revolutionized cancer treatment by effectively boosting the immune system’s ability to combat tumors. Despite their success, these therapies are only effective for a subset of cancers, likely due to the highly immunosuppressive nature of the TME.^41^^,^^42^ TME can inhibit the activity of cytotoxic immune cells, thereby diminishing the efficacy of immune checkpoint inhibitors.^43^ To overcome this limitation, strategies that enhance immune cell infiltration and activation within the TME are crucial. One promising approach involves the use of OVs engineered to express immune-activating proteins. OVs selectively infect and lyse tumor cells, releasing tumor antigens and promoting an anti-tumor immune response. Additionally, these viruses can be genetically modified to express proteins that further stimulate the immune system.^44^^,^^45^ This significant increase in the proportion of infiltrating CD8^+^ T cells and NK cells within the TME provides a theoretical foundation and ideas for combining armed oncolytic virus M1 with other immunotherapies (such as immune checkpoint inhibitors).

Previous studies delivered genes or proteins encoding GSDM to tumor tissues via intratumoral injection, using non-self-replicating armed adeno-associated viruses (rAAV) and bioorthogonal nanomaterials. The latter also faces cost and safety issues.^46^^,^^47^ Our use of intravenous injection of armed oncolytic virus M1 solves the problem of tumor targeting after tail vein injection. Although previous studies have proven the safety of the oncolytic virus M1, the long-term safety of simultaneously expressing the toxic protein GSDME remains to be observed.^6^^,^^7^^,^^8^^,^^9^^,^^10^ This is because the adverse reactions generated during traditional chemotherapy are closely related to GSDME-mediated cell pyroptosis.^15^ Despite some progress in research on GSDME and tumors, many unknowns remain regarding GSDME’s biological characteristics. TNBC is highly malignant, invasive, and prone to early recurrence, with low overall patient survival rates.^24^^,^^25^ Although immunotherapy holds a significant clinical position, TNBC is not sensitive to it, as they highly express immunosuppressive markers, resulting in very few infiltrating lymphocytes in its immune microenvironment, making it “cold” tumor.^48^^,^^49^ This study aims to activate a strong antitumor immune response by accurately delivering “missiles” (mGSDME) to TNBC using the targeting ability of OVs, inducing cell pyroptosis.

In conclusion, our study demonstrates that arming oncolytic M1 viruses with the GSDME gene significantly enhances their antitumor efficacy through the induction of pyroptosis and the promotion of immune cell infiltration in the TME. These findings highlight the potential of rM1-mGSDME_FL and rM1-mGSDME_NT as potent therapeutic agents for the treatment of TNBC and potentially other cancers. Further research into the mechanisms and broader applications of these armed viruses could pave the way for more effective cancer immunotherapies.

### Limitations of the study

Despite the promising results, there are several considerations for future studies. The specific mechanisms underlying the differential effects of mGSDME_FL and mGSDME_NT need further investigation to optimize the design of armed OVs. Additionally, while our study focused on TNBC models, it will be important to evaluate the efficacy of these armed viruses in other cancer types to determine their broader applicability. Understanding the interactions between the armed viruses and the host immune system in different tumor contexts will be crucial for the development of effective combination therapies.

## Resource availability

### Lead contact

Further information and requests for resources and reagents should be directed to the lead contact, Cheng Hu (hucheng2@mail.sysu.edu.cn).

### Materials availability

This study did not generate new unique reagents. All the cell lines used in this manuscript will be made available upon request.

### Data and code availability

#### Data

This study did not generate proteomic data, the primer sequences used for the construction of rM1-mGSDME have been listed in the key resources table.

#### Code

This paper does not report original code.

#### All other requests

Any additional information required to reanalyze the data reported will be shared by the lead contact upon request.

## Acknowledgments

This work was funded by the 10.13039/501100001809National Natural Science Foundation of China (No. 82204447); 10.13039/501100012166National Key R&D Program of China, 2021YFA0909800; the 10.13039/501100003453Natural Science Foundation of Guangdong Province (No. 2022A1515011056); the Guangzhou Science and Technology Plan Project (No. 2024A04J4711, and 2023A03J0200); and the 10.13039/501100021171Guangdong Basic and Applied Basic Research Foundation(2022B1515020056). The funders had no role in study design, data collection and analysis, decision to publish, or preparation of the manuscript.

## Author contributions

C.H., G.-m.Y., and W.-b.Z., designed the research study; X.-y.C., S.-h.L., S.W., and J.C. performed the experiments; Y.L., Y.L., and J.-k.L. analyzed the data, and C.H. wrote the paper.

## Declaration of interests

The authors declare no competing interests.

## STAR★Methods

### Key resources table

**Table:** 

REAGENT or RESOURCE	SOURCE	IDENTIFIER
**Antibodies**		

Anti-alpha Tubulin antibody [DM1A]	Abcam	Cat#ab7291
Anti-beta Actin antibody-Monoclonal	Abcam	Cat#ab8226
Anti-GAPDH antibody [6C5]	Abcam	Cat#ab8245
Anti-M1 Virus E1 Protein Monoclonal Antibody[12H]	Sino Biological	Cat#WR1-4 MM12H
Anti-DFNA5/GSDME antibody [EPR19859]	Abcam	Cat#ab215191
Caspase-3 (D3R6Y) Rabbit mAb	CST	Cat#14220S
Cleaved Caspase-3 (Asp175) (5A1E) Rabbit mAb	CST	Cat#9664S
Anti-HMGB1 antibody-Monoclonal	Abcam	Cat#ab18256
BD Pharmingen™ APC-Cy™7 Rat Anti-Mouse CD45 30-F11	BD Biosciences, USA	Cat#557659; RRID:AB_396774
BD Horizon™ BV510 Hamster Anti-Mouse CD3e 145-2C11	BD Biosciences, USA	Cat#563024; RRID:AB_2737959
BD Horizon™ BV605 Rat Anti-Mouse CD8a 53–6.7	BD Biosciences, USA	Cat#563152; RRID:AB_2738030
BD Horizon™ BV650 Rat Anti-Mouse CD4 RM4-5	BD Biosciences, USA	Cat#563747; RRID:AB_2716859
BD Horizon™ APC-R700 Rat Anti-Mouse CD44 IM7	BD Biosciences, USA	Cat#565480; RRID:AB_2739259
BD Pharmingen™ PE-Cy™7 Hamster Anti-Mouse CD69 H1.2F3	BD Biosciences, USA	Cat#552879; RRID:AB_394508
BD Horizon™ BB700 Mouse Anti-Mouse NK-1.1 PK136	BD Biosciences, USA	Cat#566502; RRID:AB_2744491
BD OptiBuild™ BB700 Hamster Anti-Mouse CD49b HMα2	BD Biosciences, USA	Cat#742140; RRID:AB_2871397
BD Horizon™ Fixable Viability Stain 620	BD Biosciences, USA	Cat#564996; RRID:AB_2869636
Anti-alpha Tubulin antibody	Arigo	Cat#ARG65693

**Bacterial and virus strains**		

M1 virus	Guangzhou Virotech Pharmaceutical Co. Ltd, Guangzhou, China	N/A
rM1-mGSDME_FL	This paper	N/A
rM1-mGSDME_NT	This paper	N/A

**Experimental models: Cell lines**		

Monkey: Vero cells	ATCC	CCL-81
Hamster: BHK-21 cells	ATCC	CCL-10
Mouse: EMT-6 cells	ATCC	CRL-2755
Mouse: EO771 cells	ATCC	CRL-3461

**Experimental models: Organisms/strains**		

Mouse: BALB/c	Guangdong Medical Laboratory Animal Center, China	Cat#GDMLAC-03

**Oligonucleotides**		

Primer for mGSDMEs: GAATGGTCCGCCGCCTTGATGT	BGI Center, Shenzhen, China	Primer ID: 210326 _002F02
Primer for M1 C to E3 Forward: ACCGGTGCACATGAAGTCAG	BGI Center, Shenzhen, China	N/A
Primer for M1 C to E3 Reverse: GTGGCGTGCACTGTTGTTAC	BGI Center, Shenzhen, China	N/A

**Recombinant DNA**		

Plasmid: pBR-M1	BGI Center, Shenzhen, China	N/A
Plasmid: pMV-MfeI-mGSDME_FL-P2A-E3-XhoI	BGI Center, Shenzhen, China	N/A
Plasmid: pMV-MfeI-mGSDME_NT-P2A-E3-XhoI	BGI Center, Shenzhen, China	N/A

**Software and algorithms**		

GraphPad Prism 8.0	GraphPad Software	https://www.graphpad.com/
CytExpert	Beckman Coulter Life Sciences	https://www.beckman.com/flow-cytometry/research-flow-cytometers/cytoflex/software
NIS-Elements	Nikon Corporation	https://www.microscope.healthcare.nikon.com/products/software/nis-elements
Image Lab Software	Bio-Rad, USA	https://www.bio-rad.com/en-jp/product/image-lab-software?ID=KRE6P5E8Z

### Experimental model and study details

#### Ethics approval

All animal treatments were performed in accordance with the Animal Research: Reporting of *In Vivo* Experiments (ARRIVE) guidelines and were approved by the Institutional Animal Care and Use Committee (IACUC) of Sun Yat-Sen University (Application No. 2022002250).

#### Cell lines

The EMT-6, EO771 and vero cell lines were purchased from the Cell Bank of the Chinese Academy of Sciences. The EMT-6 mouse triple-negative breast cancer cell line was cultured in RPMI 1640 complete medium containing 10% FBS and 1% penicillin-streptomycin. All cells were maintained at 37°C with 5% CO_2_ and 95% relative humidity.

#### Mice

A total of 24 female Balb/c immunocompetent mice, aged 6–8 weeks, were purchased from the Guangdong Medical Laboratory Animal Center. The mice were housed under specific-pathogen-free (SPF) conditions, with a controlled temperature range of 18°C–23°C and relative humidity of 40–60%. They were provided free access to standard sterile food and water and were kept on a 12-h light/dark cycle.

### Method details

#### Construction of rM1-mGSDME

The M1 plasmid vector was used as the backbone to construct the rM1-mGSDME_FL and rM1-mGSDME_NT plasmids. First, M1 viral genomic DNA vector plasmid pBR-M1, pMV-MfeI-mGSDME_FL-P2A-E3-XhoI, and pMV-MfeI-mGSDME_NT-P2A-E3-XhoI were synthesized by BGI. Target gene were digested by MfeI and XhoI(Thermo Scientic) and insert into the M1-c6v1 vector digested with the same enzymes. All the plasmids were validated by using HindIII(Thermo Scientic) to digest and check the length by electrophoresis. Digest the right plasmids by XbaI(Thermo Scientic) into linear DNA and transcript it into RNA *in vitro*(mMESSAGE mMACHINE SP6 Transcription Kit, introgen). Transfect RNA into the Vero cells using MessengerMAX transfection reagent (Thermo Fisher). Then cell culture supernatant was collected when approximately 80% of the Vero cells were infected and showed a marked CPE. Viral titers were determined using the TCID50 assay.

#### Virus production

M1 virus was produced in Vero cells cultured in VP-SFM supplemented with 10% MEM-NEAA and GlutaMAX. Following infection and the appearance of significant cytopathic effects (CPE) in approximately 90% of the cells, the supernatant was collected, centrifuged at 2000 × g for 10 min at 4°C, and stored at −80°C.

#### Tissue culture infective dose 50 (TCID50) assay

This assay was used to determine viral titers by infecting cultured cells and observing cytopathic effects (CPE). 2×10^4^ cells were seeded into 96-well plate and incubated overnight. The M1 virus was serially diluted 10-fold (10^−1^ to 10^−8^) using OptiPRO SFM medium. Each dilution was inoculated into a row of 8 wells (20 μL per well) of the 96-well plate containing cells, with two rows serving as normal cell controls. The cells were monitored daily for CPE over three days. The viral titer was calculated using the Karber method, where a dilution causing CPE in 50% of cells was used to determine the titer.

#### MTT assay

Cells in the logarithmic growth phase were seeded in 96-well plates at 4 × 10³ cells per 100 μL. The oncolytic virus M1 was serially diluted 10-fold to achieve MOIs ranging from 0.001 to 10. Each titer was tested in five replicates, with normal cell controls included. After 48 h of infection, 15 μL of MTT solution (MP Biomedicals) was added, followed by a 4-h incubation at 37°C. The succinate dehydrogenase in viable cells reduced MTT to formazan, forming purple crystals. The supernatant was removed, and 100 μL of DMSO was added to dissolve the crystals. Absorbance at 570 nm was measured using a microplate reader. Each experiment was repeated three times, and relative cell viability was calculated as: (Absorbance of treated group/Absorbance of control group) × 100%.

#### Western blots

Cells were lysed with 200 μL M-PER protein extraction reagent (containing 1× protease inhibitor and 1× phosphatase inhibitors I and II) on ice, followed by centrifugation at 12,000 ×g for 10 min at 4°C. The supernatant was used for protein quantification via the Pierce BCA Protein Assay Kit (Thermo Fisher Scientific). Samples were loaded into gel wells, and electrophoresis was conducted at 150V for 60–90 min. Proteins were transferred onto a PVDF membrane at 100V for 90 min. The membrane was washed with TBS-T and blocked with 5% milk for 1 h at room temperature. Primary antibodies, including E1 (Proteomics), GSDME (Abcam), Caspase 3 (CST), HMGB1 (Abcam), and Cleaved-caspase 3 (Abcam), were incubated with the membrane overnight at 4°C. After washing, secondary antibodies were added for 1 h at room temperature. Visualization was performed using ECL chemiluminescence, with α-tubulin as the loading control. Imaging and grayscale analysis were carried out using the Bio-Rad ChemiDoc XRS+ system and Image Lab 4.0 software.

#### Flow cytometry

On Day 13 post-tumor inoculation, tumor tissues were collected for flow cytometry. Fat, fibrous, and necrotic areas were removed, and tumors were cut into 2–4 mm pieces. The tissue was dissociated using a Tumor Dissociation Kit (Miltenyi Biotec) with the m_impTumor_02 and m_impTumor_03 programs on a homogenizer, followed by incubation at 37°C for 40 min. The cell suspension was filtered through a 70 μm mesh, centrifuged at 500 ×g for 5 min, and treated with red blood cell lysis buffer. After repeating lysis if necessary, cells were resuspended in DPBS.

A total of 3×10^6^ cells were stained with FVS 620 dye and incubated with flow cytometry antibodies, including CD45, CD3e, CD4, CD8, CD49b, or NK1.1 (all from BD Biosciences) at a 1:100 ratio for 30 min at 4°C. Flow cytometry was used to analyze the proportions of CD8^+^ cells in the CD45^+^/CD3e^+^ population and NK1.1^+^/CD49b^+^ cells in the CD45^+^/CD3e^−^ population.

#### ATP assay

The ATP assay was conducted using the Beyotime ATP Assay Kit following the manufacturer’s protocol. Cells were seeded in a 48-well plate one day prior to viral infection. The virus at 1 MOI was added to wells and thoroughly mixed. After incubation for 24 or 48 h, the positive-control cells were treated with ATP lysis buffer in wells. Then all the treatment cells supernatants were added into a 96-well plate with ATP Assay Working Solution, analyzed using a chemiluminescence plate reader (SYNERGY LX) with a 1-s measurement time and a gain of 120. The relative ATP levels were calculated based on the total cellular ATP content and plotted against the control group for comparison.

#### LDH release assay

The LDH release assay was performed using the Beyotime Lactate Dehydrogenase Cytotoxicity Assay Kit. Cells were seeded in a 48-well plate one day before viral infection. The virus at 1 MOI was added to wells. After 24 or 48 h, the positive control wells were treated with lysis buffer, mixed thoroughly, and all the cells supernatants were transferred into a 96-well plate, adding the LDH assay working buffer. The LDH levels were measured using a spectrophotometer (SYNERGY LX) at 490 nm. LDH release was calculated as a percentage of total cellular LDH and compared to the control group.

#### Tumor xenograft

100 μL of the EMT-6 cell suspension (5×10^5^ cells) was injected subcutaneously into right hind of each mouse. On the 6 days post-tumor inoculation (Day 6), when the tumor volume reached approximately 50 mm^3^ (length × width × width/2), the mice were randomly divided into four groups and administered the following treatments via a single tail vein injection: (1) Control group: 300 μL PBS; (2) Wild-type M1 group: 300 μL of M1 oncolytic virus at a titer of 1.0 × 10^7^ PFU/mL; (3) rM1-mGSDME_FL group: 300 μL of rM1-mGSDME_FL oncolytic virus at a titer of 1.0 × 10^7^ PFU/mL; (4) rM1-mGSDME_NT group: 300 μL of rM1-mGSDME_NT oncolytic virus at a titer of 1.0 × 10^7^ PFU/mL. Tumor size and mortality were recorded every 3 days. Humane endpoints were defined as a tumor length exceeding 20 cm or a tumor volume exceeding 2500 mm^3^, at which point mice were euthanized.

### Quantification and statistical analysis

Quantitative data were shown as mean ± standard deviation. Statistical analysis was performed using t-tests or one-way ANOVA with Dunnett’s multiple comparison test. Tumor growth curves were analyzed using variance analysis for repeated measures, and survival curves were analyzed using the log rank test. Statistical significance was indicated as follows: n.s. (not significant); ∗ (*p* < 0.05); ∗∗ (*p* < 0.01); ∗∗∗ (*p* < 0.001).
